# Environmental effects on brain functional networks in a juvenile twin population

**DOI:** 10.1038/s41598-023-30672-2

**Published:** 2023-03-09

**Authors:** Emma Tassi, Eleonora Maggioni, Maddalena Mauri, Corrado Fagnani, Nivedita Agarwal, Anna Maria Bianchi, Maria A. Stazi, Maria Nobile, Paolo Brambilla

**Affiliations:** 1 Department of Electronics, Information and Bioengineering, Politecnico di Milano, Milan, Italy; 2 Department of Neurosciences and Mental Health, Fondazione IRCCS Ca’ Granda Ospedale Maggiore Policlinico, Milan, Italy; 3 Child Psychopathology Unit, Scientific Institute, IRCCS Eugenio Medea, Bosisio Parini, Italy; 4 PhD Program in Neuroscience, School of Medicine and Surgery, University of Milano-Bicocca, Bosisio Parini, Italy; 5 Centre for Behavioural Sciences and Mental Health, Istituto Superiore di Sanità, Rome, Italy; 6 Neuroimaging Lab, Scientific Institute, IRCCS E. Medea, Bosisio Parini, Italy; 7 Department of Pathophysiology and Transplantation, University of Milan, Milan, Italy

**Keywords:** Genetics, Neuroscience

## Abstract

The brain’s intrinsic organization into large-scale functional networks, the resting state networks (RSN), shows complex inter-individual variability, consolidated during development. Nevertheless, the role of gene and environment on developmental brain functional connectivity (FC) remains largely unknown. Twin design represents an optimal platform to shed light on these effects acting on RSN characteristics. In this study, we applied statistical twin methods to resting-state functional magnetic resonance imaging (rs-fMRI) scans from 50 young twin pairs (aged 10–30 years) to preliminarily explore developmental determinants of brain FC. Multi-scale FC features were extracted and tested for applicability of classical ACE and ADE twin designs. Epistatic genetic effects were also assessed. In our sample, genetic and environmental effects on the brain functional connections largely varied between brain regions and FC features, showing good consistency at multiple spatial scales. Although we found selective contributions of common environment on temporo-occipital connections and of genetics on frontotemporal connections, the unique environment showed a predominant effect on FC link- and node-level features. Despite the lack of accurate genetic modeling, our preliminary results showed complex relationships between genes, environment, and functional brain connections during development. A predominant role of the unique environment on multi-scale RSN characteristics was suggested, which needs replications on independent samples. Future investigations should especially focus on nonadditive genetic effects, which remain largely unexplored.

## Introduction

Recent technical and methodological advancements in functional Magnetic Resonance Imaging (fMRI) field have enabled an accurate characterization of functional brain networks during resting state, leading to important information about the brain pathophysiology. At rest the human brain is organized in multiple large-scale networks, called resting-state networks (RSNs), resulting from the synchronous activation of distant brain regions involved in processes like vision, audition, motor planning, memory, and attention^1–4^. Independent component analysis (ICA) findings demonstrated high spatial consistency of the RSNs across subjects^2,3,5,6^. Their reproducibility was confirmed by seed-based functional connectivity (FC) studies, which identified sets of nodes being consistently organized into functional modules^7,8^. Of note, connectivity’s alterations within and among the RSNs were observed in different neuropsychiatric disorders^9,10^. Shifting from a group-level to a subject-level perspective, recent fMRI findings showed the existence of individual functional brain network characteristics that remain unique and stable across years^11–13^. Accordingly, subject-specific functional brain network topography seems predictive of behavioral phenotypes^14^.

In this respect, how developmental changes result in individualized FC signatures is still debated. Studying the developmental determinants of FC might inform on individual differences in behavior, giving insight into complex mental disorders’ etiology^15^. Although RSNs result to be largely established early in development, passing from childhood to adolescence they exhibit a shift from a local (segregated) to a more distributed (integrated) topological organization^16^. Individualized FC fingerprints progressively emerge from childhood to early adulthood^17^, arising from influences of genetics, environment, and their interaction that remain largely unexplored^18^.

Twin studies provide an excellent platform for the estimation of developmental determinants and heritability on RSNs, by considering that monozygotic (MZ) twins carry identical genomes, while dizygotic (DZ) twins share approximately 50% of the genome. Within this context, ACE and ADE twin models compare the intra-pair correlation between monozygotic (MZ) and dizygotic (DZ) twin pairs to estimate the proportion of phenotypic variance explained by additive genetics (A), unique environment (E), and common environment (C) or nonadditive genetics (D) influences, respectively. Thus, twin design applied on resting state fMRI (rs-fMRI) data allows the most accurate estimation of genetic and environmental effects on intrinsic FC patterns^19^, allowing the characterization of inter-individual neurodevelopmental differences.

Up until now, just a few studies investigated the determinants of inter-subject RSN variability, especially during the neurodevelopment^20–24^. Their results suggest complex scale-, region-, and feature-specific relationships between genetics and RSNs, which do largely vary from after birth^20^, through childhood and adolescence^22,23^ and up to young adulthood^21^. Predominant genetic contributions were reported on regional FC metrics during the first two years of life^20^, as well as environmental effects on selective frontolimbic pathways during childhood and adolescence^19,20^. Findings on global FC metrics suggest significant genetic influences on global brain efficiency since childhood^24^. Moderate heritability of this and other common graph metrics was observed by Sinclair et al*.*^21^ in a large sample of twins aged between 18 and 30 years. Interestingly, heritability estimates were found to depend on network connection densities, with greater heritability values associated with network metrics at lower connection densities.

Current evidence suggests that the brain’s FC metrics are largely heritable in selected time windows from infancy onwards, but dynamic environmental influences on selective RSNs seem to occur as well. The heterogeneity of the available studies arises from methodological differences that span from preprocessing to the employed statistical twin design, resulting in fragmented knowledge. Thus, there remains the urgent need to better understand intrinsic FC metrics’ determinants throughout development.

In this context, using a whole-brain, multi-model, multi-scale, and multi-feature approach, the present twin study aimed to cross-sectionally explore the genetic and environmental contributions on functional connectome during a long and delicate developmental phase, from late childhood to early adulthood. A rs-fMRI dataset obtained from 43 twin pairs aged between 10 and 30 years was used to extract FC features at multiple spatial scales, from each network link to the whole-brain network. Although our small sample impeded a Structural Equation Model (SEM) analysis, we tested the applicability of twin models and, when possible, decomposed the feature variance into genetic and environmental components.

Based on limited evidence to date, we hypothesized that the majority of FC metrics at different network scales would be under stronger genetic control compared to shared environmental control. In view of the spatially heterogeneous individual FC variability^25^, we expected non-uniformly distributed genetic-environmental influences on local FC patterns. Furthermore, as heritability estimates may vary according to the connection densities of the network^21^, we expected greater genetic effects in relevant long-range functional pathways compared to small-network short-scale pathways.

## Materials and methods

### Study population

The dataset employed in the present study was collected from 50 pairs of twins (composed of 42 males and 58 females, mean age 18.5 years, age range 10–30 years) at the Scientific Institute IRCSS E. Medea of Bosisio Parini (Lecco, Italy). The twin study, which was conducted in collaboration with the Italian Twin Registry^26^, was aimed to explore the genetic and environmental contributions to brain development and behavior. To this end, socio-demographic, psychopathological, behavioral, cognitive, genetic, and neuroimaging information were collected from all participants at a single time point. Here, only socio-demographic and neuroimaging information was used to pursue the study’s primary objective. Exclusion criteria were intelligence quotient (IQ) < 70 based on Wechsler intelligence scales for adults/children (WAIS/WISC)^27,28^ diagnosis of autism spectrum disorder, epilepsy or other neurological disorders, history of head trauma, severe visual, auditory, or language comprehension deficits, number of gestation weeks < 34. The research protocol was approved by the competent Research Ethical Committee in accordance with the 2013 Fortaleza version of the Helsinki Declaration and subsequent amendments. A written informed consent to the study was obtained by all participants of legal age, or their parents in the case of minors. In view of the sexual dimorphism of brain morphology and function, the rs-fMRI data processing was performed only on 46 same-sex twin pairs, of which 19 pairs were monozygotic (MZ) (10 male pairs, 9 female pairs, 16.7 ± 5.25 years) and 27 pairs were dizygotic (DZ) (9 male pairs, 18 female pairs, 19.3 ± 6.59 years). After quality check (described in the “fMRI pre-processing” section), 3 additional pairs were excluded from the statistical analyses, resulting in a dataset of 43 twin pairs (25 DZ and 18 MZ).

### MRI acquisition protocol

Structural and functional MRI data were acquired at the IRCCS E. Medea using a 3T scanner (Philips Achieva, Best, The Netherlands) equipped with a 32-channel head coil. rs-fMRI volumes (n = 200) were obtained using a T2*-weighted echo planar imaging (EPI) sequence with the following parameters: repetition time (TR) = 2500 ms, echo time (TE) = 35 ms, flip angle = 8°, 30 axial slices with no gap, in-plane matrix size = 128 × 128, voxel size = 1.8 mm × 1.8 mm × 3 mm. During the rs-fMRI session, the subjects were asked to keep their eyes closed, not to think about anything in particular, and not to fall asleep. The morphological reference for the fMRI results was provided by a 3D T1-weighted turbo field echo (TFE) SENSE sequence with TR = 8.3 ms, TE = 3.8 ms, flip angle = 8°, 190 axial slices with no gap, in-plane matrix size = 240 × 240, voxel size = 1 mm × 1 mm × 1 mm.

### MRI analyses

The MRI data pre-processing, connectivity feature extraction, and statistical analyses were mainly performed in Matlab R2019b (The MathWorks, Inc.) using the open-source Statistical Parametric Mapping (SPM) software (version 12, https://www.fil.ion.ucl.ac.uk/spm/software/spm12/)^29^, the open-source Brain Connectivity Toolbox (BCT) (https://sites.google.com/site/bctnet/)^30^, and in-house Matlab scripts including functions from the Statistics and Machine Learning toolbox. The FSL software (version 6, https://fsl.fmrib.ox.ac.uk/fsl/fslwiki/)^31^, was also used for a pre-processing step. The fMRI processing pipelines are illustrated in schematic form in the Supplementary Figure S1.

#### fMRI pre-processing

The raw fMRI volumes from each subject were imported in SPM12 and spatially realigned to the subject’s first volume using the least squares approach followed by a rigid body transformation. The subject’s structural T1-weighted image was co-registered to the mean fMRI image through an affine transformation, corrected from intensity biases, and segmented into different tissue types. The forward deformation field parameters estimated in the latter step were used for normalization of the fMRI volumes from the subject’s native space to the standard Montreal Neurological Institute (MNI) space. The realigned and normalized fMRI volumes were smoothed using a 3D Gaussian kernel filter with Full Width at Half Maximum (FWHM) equal to 6 mm and imported in the FSL software. Here, the pre-processed fMRI volumes from each subject were temporally high-pass filtered (cut-off frequency of 0.01 Hz) and entered into a single-subject spatial ICA decomposition using the FSL Multivariate Exploratory Linear Optimized Decomposition into Independent Component (MELODIC) toolbox (https://fsl.fmrib.ox.ac.uk/fsl/fslwiki/MELODIC). Artefactual independent components (ICs) were marked using a semi-automatic spatiotemporal tool recently published by our research group and subsequently regressed out from the fMRI volumes^32^. In our study, the choice to use the proposed tool was motivated by its good performances in the detection of noise-related ICs in rs-fMRI data (> 80% of accuracy, sensitivity, and specificity in 32). The tool marks an unknown IC as either artefactual or physiological based on (i) the spatial correlation between its spatial map and those from labeled artefactual ICs, (ii) the proportion of high-frequency content (> 0.1 Hz) in its time series, as assessed via a relative power spectral analysis. ICs with values of spatial correlation or high-frequency power higher than predefined thresholds (identified as optimal in^32^) were marked as artefactual. The results from the automatic IC labeling were subjected to a final careful inspection, which was followed by the removal of noise-related ICs.

The resulting fMRI volumes were subjected to the connectivity and statistical analyses described in the next sections.

In a final quality check, the subjects with a percentage of noise-related ICs > 75% and their siblings were excluded from the analyses, resulting in a dataset composed of 43 twin pairs.

The overall quality of the fMRI dataset was assessed by monitoring the extent of movement artefacts before and after the denoising steps. Specifically, the extent of motion in the original fMRI dataset was measured using framewise displacement (FD), whereas the effects of denoising on motion artefacts were quantified using the FD-DVARS metric, which was compared from before to after pre-processing via paired t-tests.

In the original fMRI dataset, the average FD across included subjects was 0.41 mm (± 0.76 mm) below the commonly used FD censoring threshold of 0.5 mm. Furthermore, our pre-processing pipeline resulted in a significant reduction of FD-DVARS (*p* < 0.001), which on average decreased from 0.78 (± 0.20) to 0.64 (± 0.21) before ICA, up to 0.60 (± 0.21) after ICA.

#### Seed-based FC analysis

The SPM Marsbar toolbox (version 0.44, http://marsbar.sourceforge.net/) was used to obtain the parcellation of the subjects’ fMRI volumes in $N$ = 90 regions of interest (ROIs) of the Automated Anatomical Labeling (AAL) atlas^33^, representing the nodes for the functional connectivity (FC) analysis. We selected all AAL ROIs except from the ones located in the cerebellum and vermis, which were not covered by the fMRI volumes in some or all subjects.

For each subject, we extracted the mean BOLD time series of the voxels within each ROI (node). Using Matlab in-house scripts, instantaneous statistical dependencies among ROIs were assessed by computing the Pearson correlation coefficients between the BOLD time series of each pair of ROIs, resulting in a $N\times N$ FC adjacency matrix for each participant, whose elements represent the pairwise cross-correlation between the BOLD time series of the corresponding ROIs. Only the functional connections corresponding to significant Pearson correlation values (*p* < 0.05) were considered, by setting to zero the non-significant ones. The resulting subject-level FC matrices (either weighted or binarized using an arbitrary positive 0.5 threshold) were further analysed to extract FC features of interest.

#### FC feature extraction

Brain network properties at multiple spatial scales, i.e., at the link and node levels and in the entire network ($N$ = 90 AAL ROIs), were extracted from the subject-level FC matrices. Node-level and network-level topological features were extracted using graph theory functions from the BCT^30^. For each ROI (node), the node-level degree, local efficiency, clustering coefficient, and betweenness centrality were computed from the binarized FC matrix, whereas the node-level strengths of positive and negative weights were computed from the weighted FC matrix. The binarized FC matrix was also used to extract the whole-brain network-level global efficiency, characteristic path length, degree, density, and Louvain modularity. The detailed description of each FC feature is reported in the Supplementary Table S1 and Supplementary Table S2.

#### Statistical twin analyses

The link-, node-, and network-level FC features (detailed in the previous section) were correlated between twins, and intra-pair correlations were compared between MZ and DZ pairs to disentangle the relative contributions of genetic and environmental factors, as described in the following sections.

#### Intra-pair FC correlation

For each FC metric, separately for MZ and DZ twin pairs, we computed the linear partial correlation coefficient between the selected feature values obtained from Twin#1 and Twin#2 in each pair, at net of the effects of age and sex. As a result, we obtained zygosity-specific correlations (${r}_{MZ}$ and ${r}_{DZ}$) for the link-level weights ($n$=4500, elements $(i, j)$ of the FC matrix, $i=1:(N-1), j=\left(i+1\right):N$), as well as the node-level ($n$=540) and the network-level ($n$=5) parameters. In each case, the ${r}_{MZ}$ and ${r}_{DZ}$ values were then compared to verify the applicability of genetic modelling and, if that was the case, to select the optimal model to be used, as described in the following Section.

#### Genetic and environmental influences on FC features

**Figure 1:**
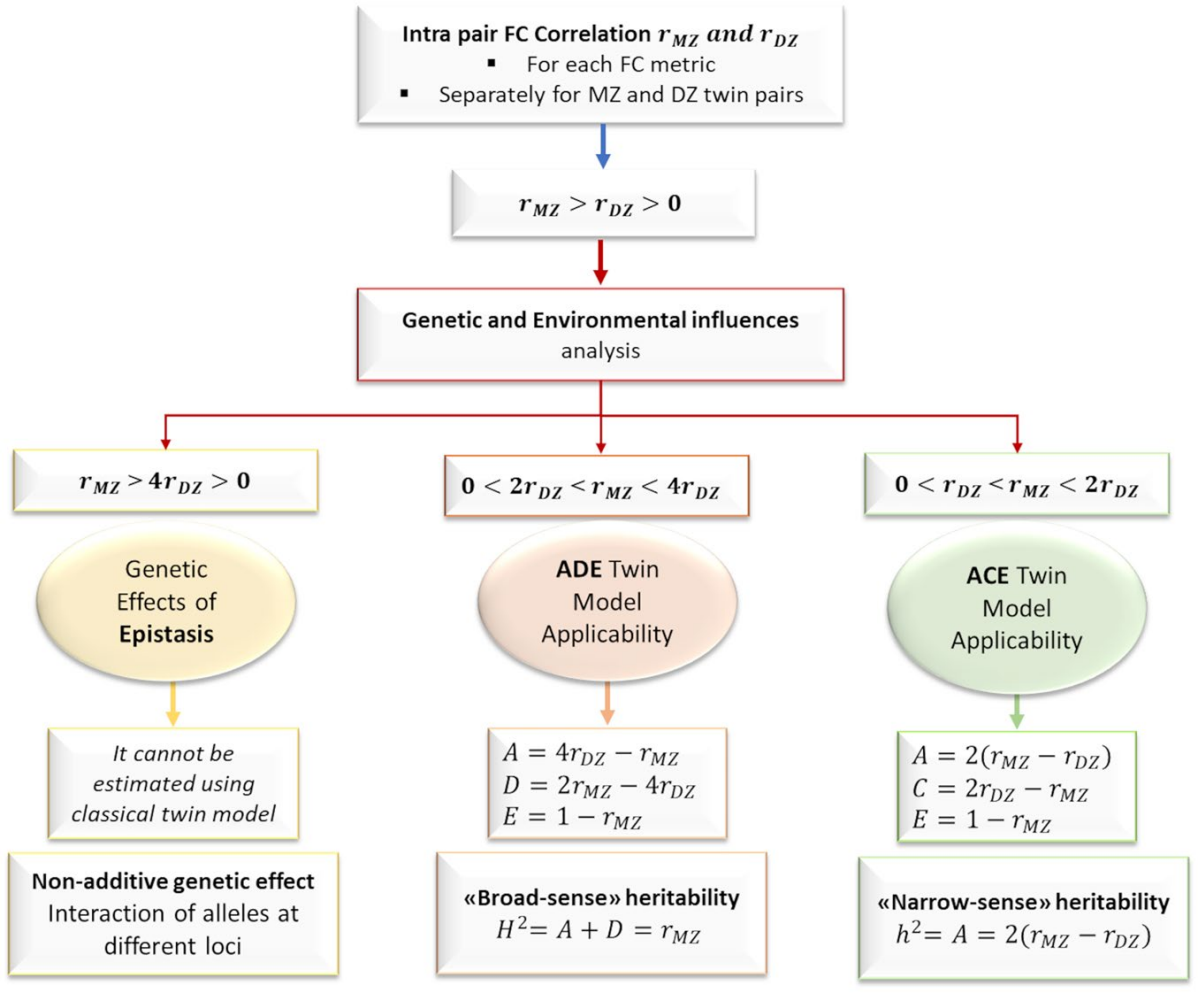
Schematic illustration of genetic and environmental analysis pipeline. For each FC metric, separately for MZ and DZ twin pairs, we computed the linear partial correlation coefficient between the selected feature values obtained from each twin in each pair, at net of the effects of age and sex. We obtained zygosity-specific correlations (${r}_{MZ}$ and ${r}_{DZ}$) for each FC metric at brain-, node- and link-level. Genetic and environmental analysis was performed on the FC metrics associated with ${r}_{MZ}>{r}_{DZ}>0$. Three main scenarios were distinguished based on $\frac{{r}_{MZ}}{{r}_{DZ}}$ ratio: ADE or ACE twin model applicability and the analysis of nonadditive genetic effect of epistasis. The estimate of “broad-sense” or “narrow-sense” heritability was extracted for the FC metrics that verified respectively ADE or ACE twin model applicability condition. FC: functional connectivity. MZ: monozygotic. DZ: dizygotic. ${\mathrm{r}}_{\mathrm{DZ}}$: intra-pair phenotypic correlation in DZ pairs. ${r}_{MZ}$: intra-pair phenotypic correlation in MZ pairs.

The analysis of genetic and environmental influences was performed on the FC features characterized by 1) ${r}_{MZ}$ > 0 and ${r}_{DZ}$ $>0$, 2) ${r}_{MZ}$ > ${r}_{DZ}$. The complete twin statistical modelling pipelines used to extract genetic and environmental components are illustrated in schematic form in Fig. 1.

Three main scenarios of genetic and environmental contributions were distinguished based on the ${r}_{MZ}/{r}_{DZ}$ ratio^34,35^. These scenarios involve three of the following four possible sources of influence: (i) additive genetic source (A), representing the effects of all alleles that influence the phenotype; either (ii) nonadditive genetic source (D), representing interactions between alleles at the same locus (dominance) or at different loci (epistasis), or (iii) shared environmental source (C), representing exposure effects that are common to family members (e.g., family exposures during infancy and childhood); (iv) unique environmental source, representing individual-specific exposure effects (e.g., lifestyles, infections and diseases, traumatic events).

When the condition ${r}_{MZ}>4{r}_{DZ}>0$ was satisfied, the FC parameter under analysis was assumed to be influenced by nonadditive genetic epistatic effects. These effects cannot be resolved using classical twin designs, that is, they cannot be estimated as a linear function of the ${r}_{MZ}$ and ${r}_{DZ}$ values associated with the parameter.

When the condition $0<{2r}_{DZ}<{r}_{MZ}<4{r}_{DZ}$ was verified, both genetic (additive and dominant) and unique environmental factors were hypothesized to affect the FC feature. These contributions were modelled using the ADE model, composed of additive (A) and dominant (D) genetic effects, as well as unique environmental influences (E). The proportions of variance in the FC feature that are due to these three factors were estimated as: $A=4{r}_{DZ}- {r}_{MZ}$, $D=2{r}_{MZ}-4{r}_{DZ}$ and $E=1-{r}_{MZ}$. A “broad sense” estimate of heritability was obtained as the sum of the A and D coefficients: ${H}^{2}=$ $A+D={r}_{MZ}$, which thus provides information on the proportion of variance in the phenotype that is explained by total (additive and dominant) genetic variance.

When the condition $0<{r}_{DZ}<{r}_{MZ}<2{r}_{DZ}$ was verified, we considered the contributions of additive genetic (A) and shared (C) and unique (E) environmental factors on the FC parameter, which were estimated using the ACE model as: $A=2({r}_{MZ}-{r}_{DZ})$, $C=2{r}_{DZ}-{r}_{MZ}$ and $E=1-{r}_{MZ}$. A “narrow sense” estimate of heritability was provided by the coefficient A: ${h}^{2}=A=2({r}_{MZ}-{r}_{DZ})$, which thus quantifies the proportion of phenotypic variance that is accounted for by additive genetic variance.

For all link-, node-, and network-level FC features, regardless of the most suitable genetic model, significant differences between ${r}_{MZ}$ and ${r}_{DZ}$ values were assessed based on their Fisher’s Z-transforms, setting the significance threshold to *p* = 0.05. Multiple comparisons’ correction was performed using the Bonferroni method by considering as N the number of comparisons per condition (e.g., epistasis, ACE, ADE). Both uncorrected (p_unc_ < 0.05) and Bonferroni corrected (p_Bonf_ < 0.05) results will be shown. Furthermore, in the main resting state networks (RSNs, described in Table 5), we computed the percentage of network connections for which epistatic effects or genetic plus environmental effects—as explained by ACE or ADE twin models—were detected (post-hoc RSN-level analysis). The list of AAL atlas regions included in each RSN under analysis is reported in the Supplementary Table S3.

## Results

This section reports the twin sample characteristics and the estimated genetic and environmental effects on multi-scale FC features, the latter described only link-level, node-level, and post-hoc RSN-level results are described.

### Sample

The original socio-demographic and rs-fMRI dataset was collected from 50 pairs of twins. After exclusion of twin pairs with sex differences (n = 4) or low-quality rs-fMRI data during the noise-related ICs quality check step (n = 3), the resulting sample of 43 twin pairs, including 25 DZ pairs and 18 MZ pairs, was selected for subsequent statistical analyses (schematized in Fig. 1). A power analysis based on Monte Carlo simulations (n = 10,000) showed that our sample enabled the detection of a strong broad-sense heritability (60%) against the null hypothesis of no heritability with 86.70% power (95% confidence interval 84–89%).

The selected sample’s characteristics are detailed in Table 1. Age, sex, and behavioral characteristics based on the Child/Adult Behavior CheckList (CBCL/ABCL) were comparable between MZ and DZ twin pairs.

**Table 1:** Demographic and behavioral information of the sample.

	MZ	DZ	Stats
N twins	36	50	–
N Females, N Males	18, 18	34, 16	${\chi }^{2}$=1.418, * p* = 0.234 (pair-based)
Age [years]	17.012 ± 5.185	20.094 ± 6.261	T = 1.708, * p* = 0.096 (pair-based)
ABCL/CBCL TOT	49.41 ± 7.81	49.13 ± 7.79	T = 0.155, * p* = 0.877 (subject-based)
ABCL/CBCL INT	51.735 ± 8.426	50.727 ± 8.287	T = 0.529, * p* = 0.599 (subject-based)
ABCL/CBCL EXT	47.000 ± 9.531	48.977 ± 8.812	T = 0.948, * p* = 0.346, (subject-based)

For continuous variables, means ± standard deviations are reported. MZ: monozygotic; DZ: dizygotic; T: T-statistics; χ^2^: χ^2^ statistics; p: p-value; ABCL/CBCL: Adult/Child Behaviour CheckList; TOT: total score; INT: internalization score; EXT: externalization score.

### Link-level FC metrics

**Figure 2:**
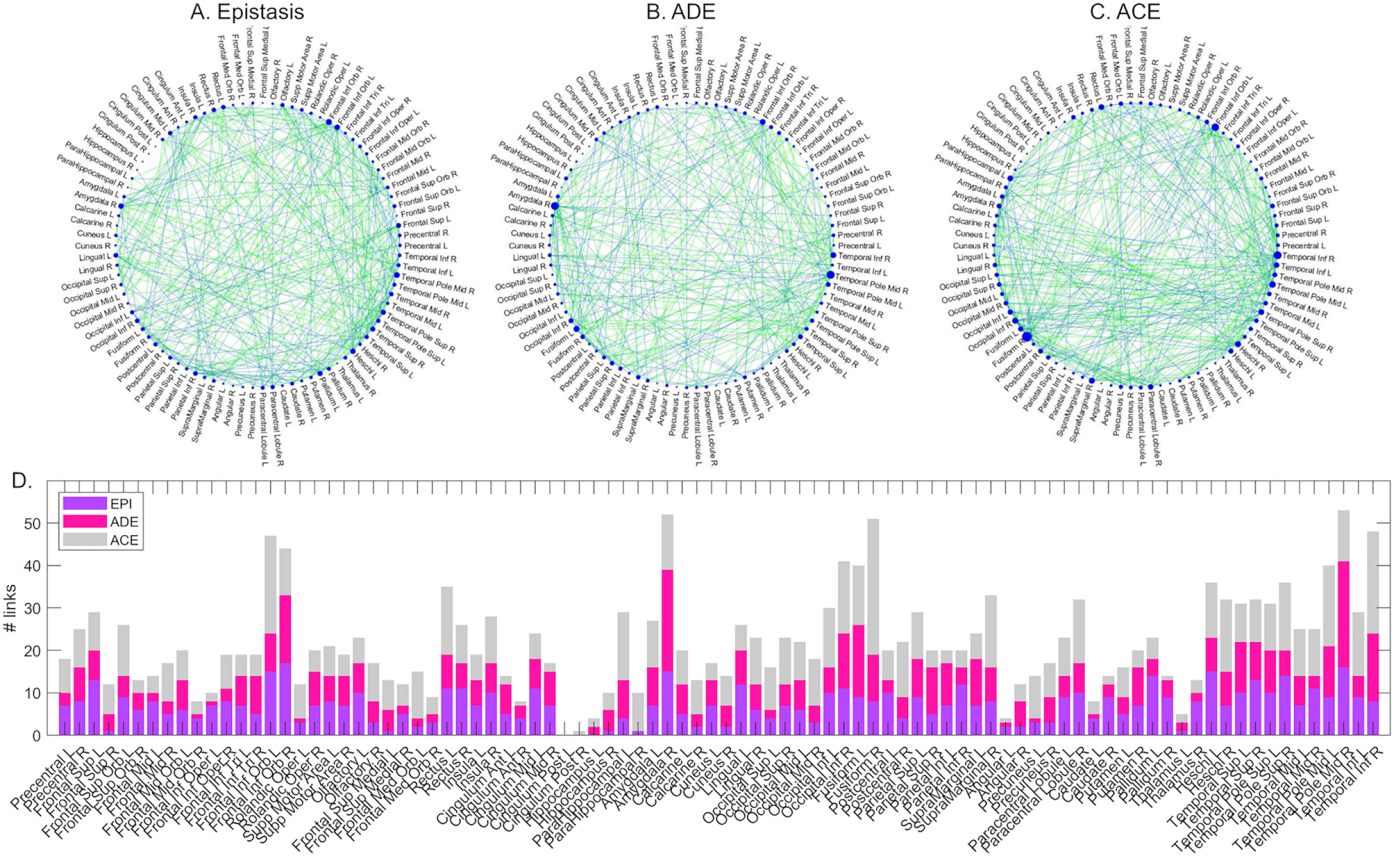
Twin models on link-level FC metrics. A-C. Graphs representing the brain functional connections influenced by epistasis (**A**), ADE factors (**B**), and ACE factors (**C**). Intra-hemispheric and inter-hemispheric connections are represented in blue and green, respectively. The size of each ROI (node) in the graph is proportional to the number of ROI connections influenced by the corresponding factor. D. Stacked bar plot representing the numbers of links per node influenced by epistasis (violet), ADE factors (magenta), and ACE factors (grey). FC: functional connectivity. ROI: region of interest. Panels were created with Matlab R2019b software (http://www.mathworks.com), assembled with Microsoft Power Point software (https://www.microsoft.com/it-it/microsoft-365/powerpoint) and exported using GIMP v.2–10 (https://www.gimp.org/).

**Figure 3:**
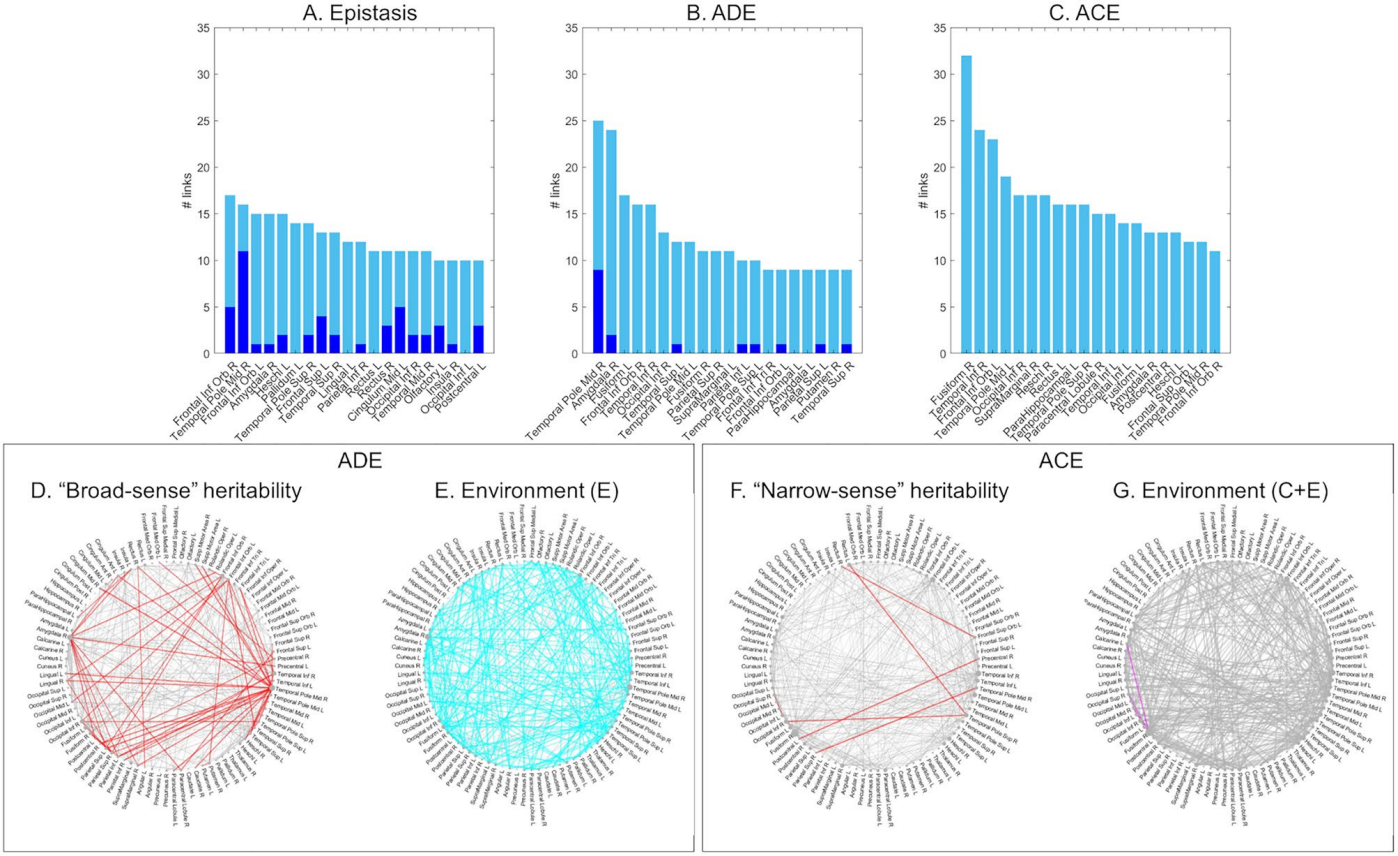
ROI sorting based on link-level effects. A-C. Bar plots representing in light blue the number of functional connections influenced by epistasis (**A**), ADE factors (**B**), and ACE factors (**C**) in descending order in the 20 top ranking ROIs. Network links with significant correlation differences (*p* < 0.05) extracted from Fisher’s Z statistics between MZ and DZ pairs are highlighted in dark blue in panels (**A**, **B**). (**D**–**E**) Functional connections suitable for ADE model weighted for their genetic (**D**) and environmental (**E**) contributions. In D, links with “broad-sense” heritability > 50% are highlighted in red. In E, links with unique environmental effects (**E**) > 50% are highlighted in cyan. F-G. Functional connections suitable for ACE model weighted for their genetic (**F**) and environmental (**G**) contributions. In G, links with “narrow-sense” heritability > 50% are highlighted in red. In G, links with common environmental effects (C) > 50% are highlighted in magenta. Panels were created with Matlab R2019b software (http://www.mathworks.com), assembled with Microsoft Power Point software (https://www.microsoft.com/it-it/microsoft-365/powerpoint) and exported using GIMP v.2–10 (https://www.gimp.org/).

The FC links associated with (1) epistatic effects, (2) additive/dominant genetic and unique environmental effects (ADE model factors), or (3) additive genetic and common/unique environmental effects (ACE model factors) are highlighted in Fig. 2A–C graphs. In these graphs, the higher is the dimension of a region of interest (ROI, node), the higher is the number of functional links involving that ROI and showing the corresponding effect. The stacked bar plots in Fig. 2D show, for each ROI, the number of links characterized by the three effects. The stacked bar plots in Fig. 3A–C show, for each effect, the number of links per ROI with the specified effect in the top 20 ROIs in descending order. The Fig. 3D–G graphs illustrate the links suitable for ADE/ACE modelling weighted according to the relative genetic and environmental contributions.

#### Epistasis

The 7.97% of all functional connections were found to depend on nonadditive genetic effects from alleles at multiple loci, that is, on epistatic effects. Notably, these links were balanced between inter-hemispheric (51.10%) and intra-hemispheric (48.90%) ones. As shown in Fig. 3A, depending on the ROI, epistatic effects were present in a number of ROI connections ranging from 0 to 17 (19.10%). No epistatic effects were observed in the links involving the bilateral posterior cingulate cortex and left hippocampus, whereas bilateral inferior orbitofrontal cortex, right middle temporal pole, right amygdala, left Heschl’s gyrus, and left pallidum were characterized by more than 15% of connections with epistatic effects.

The comparison between ${r}_{MZ}$ and ${r}_{DZ}$ values based on Fisher’s Z-transform revealed significant differences, even if at the uncorrected level (p_unc_ < 0.05), in 13.79% of the network links showing epistatic effects (highlighted in blue for the top 20 ROIs in Fig. 3A). Right middle temporal pole was the ROI with the highest number of links being significantly more correlated in MZ twins than in DZ twins (n = 11). The highest correlation difference was observed in the connection between right middle temporal pole and right precentral gyrus (Z = 2.94, *p* = 0.0016).

#### ADE model

The functional connections influenced by both additive and dominant genetic factors accounted for 7.14% of the total number of links, balanced between intra-hemispheric (48.95%) and inter-hemispheric (51.05%) ones. The number of links per ROI influenced by ADE factors ranged from 0, in the bilateral posterior cingulate cortex and right parahippocampal gyrus, to 25 (28.09%), in the right middle temporal pole (Fig. 3B). The right amygdala showed the second highest percentage of links suitable for ADE modelling (n = 24, 26.97%).

Based on the Fisher’s Z statistics, significant correlation differences between MZ and DZ pairs emerged, only at the uncorrected level, in 4.20% of these links (p_unc_ < 0.05, highlighted in blue for the top 20 ROIs in Fig. 3B). Again, the right middle temporal pole showed the highest number of connections for which ${r}_{MZ}$ was significantly higher than ${r}_{DZ}$ (n = 9). The most significant Z statistics (i.e., correlation difference) was observed in its connection with the right middle cingulate cortex (Z = 2.59, *p* = 0.005).

The “broad-sense” heritability estimates (${H}^{2}=A+D$) for these links indicated an average predominance of environmental effects (E = 63.38 ± 16.19%) over genetic ones (${H}^{2}$= 36.62 ± 16.19%). The links with ${H}^{2}$ > 50% accounted for 18.88% of the total (highlighted in red in Fig. 3D), whereas the remaining links showed predominant E effects (highlighted in cyan in Fig. 3E). The ${H}^{2}$ maximum was observed in the connection between right middle temporal pole and right middle cingulate cortex (corresponding to the peak Z statistic).

#### ACE model

The links meeting the ACE model criteria represented 9.61% of the total. Among them, intra-hemispheric and inter-hemispheric links were roughly balanced, accounting for 48.83% and 51.17%, respectively. The number of ROI connections affected by ACE factors varied from 0 in the left posterior cingulate cortex to 32 (35.96%) in the right fusiform gyrus (Fig. 3C). Proportions of these links higher than 20% were also observed in the right inferior temporal gyrus, left inferior orbitofrontal cortex, and left middle temporal pole.

The Fisher’s Z-statistics comparison showed no significant correlation differences between MZ and DZ pairs in any of the links suitable for ACE modelling. Accordingly, the “narrow-sense” heritability estimates for these links, corresponding to the additive (A) genetic effects, indicated an average predominance of environmental influences (C + E = 81.91 ± 13.23%) over genetic ones (${h}^{2}$= 18.09 ± 13.23%). Only five links (1.56% of the ACE links) were characterized by ${h}^{2}$>50% (highlighted in red in Fig. 3F).

Although shared environmental effects were lower than unique environmental ones (C = 12.29 ± 10.52%, E = 69.62 ± 14.98%), they accounted for more than 50% of the FC variance in three connections (0.78% of the ACE links, highlighted in magenta in Fig. 3G). All these links involved the right fusiform gyrus, with (1) left calcarine cortex (C = 64.00%), (2) right superior occipital cortex (C = 53.91%), and (3) right middle occipital cortex (C = 55.20%).

### Node-level FC metrics

The locations of node-level FC features (degree, local efficiency, clustering coefficient, betweenness centrality, strength of positive weights, and strength of negative weights) influenced by (1) epistatic effects, (2) ADE model factors, or (3) ACE model factors are listed in the following sections. ${r}_{MZ}$ and ${r}_{DZ}$ values for all these node-level features are illustrated in the Supplementary Figure S2.

#### Epistasis

Depending on the FC metric, specific ROIs were found to be influenced by epistatic effects. The most influenced features were the nodal degree and the nodal strength of positive weights, for which epistatic effects were observed in 11.11% and 10.00% of the network ROIs, respectively. Conversely, the local efficiency feature showed these effects only in 3.33% of the ROIs. For each nodal parameter, the ROIs influenced by epistatic effects are summarized in Table 2, together with the corresponding Fisher’s Z statistics and related p values. Specifically, all the ROIs associated with both non-significant and significant p-value estimates are reported, with Bonferroni corrected results in bold. Significant correlation differences between MZ and DZ pairs (*p* < 0.05) emerged for (1) the strength of positive weights in right inferior orbitofrontal cortex, (2) the strength of negative weights in the right inferior temporal gyrus, (3) the degree of connections of right amygdala, and (4) the clustering coefficient of right middle orbitofrontal cortex. Of note, correlation differences that emerged for the strength of negative weights in right inferior temporal gyrus remained significant after Bonferroni correction (Z = 4.25, p_Bonf_ < 0.001).

**Table 2:** Location and statistics of epistatic effects on node-level FC metrics, reported for each ROI that result significantly and non-significantly influenced by epistatic effect.

Node-level FC metric	ROIs	Z-statistics	p-values
Strength of positive weights	Frontal Inf Orb L	Z = 1.2159	n.s
	Frontal Inf Orb R	Z = 1.7323	*p* = 0.0416
	Amygdala, R	Z = 1.4793	n.s
	Occipital Inf L	Z = 0.3426	n.s
	Paracentral Lobule L	Z = 0.9264	n.s
	Paracentral Lobule R	Z = 0.9295	n.s
	Heschl L	Z = 0.8185	n.s
	Temporal Pole Sup R	Z = 1.2350	n.s
	Temporal Pole_Mid L	Z = 0.4449	n.s
Strength of negative weights	Cingulum Ant R	Z = 1.1804	n.s
	Cingulum Mid L	Z = 1.3585	n.s
	Cingulum Mid R	Z = 0.8367	n.s
	Parietal Inf L	Z = 0.5618	n.s
	Precuneus L	Z = 0.6659	n.s
	Temporal_Inf_R	Z = 4.2472	***p* < 0.001**
Degree	Precentral R	Z = 1.0946	n.s
	Olfactory L	Z = 0.8336	n.s
	ParaHippocampal R	Z = 0.2017	n.s
	Amygdala R	Z = 2.0414	*p* = 0.0206
	Lingual R	Z = 0.9892	n.s
	Fusiform L	Z = 0.4646	n.s
	Postcentral R	Z = 0.7843	n.s
	Paracentral Lobule L	Z = 1.2837	n.s
	Temporal Mid L	Z = 0.4837	n.s
	Temporal Inf R	Z = 0.9516	n.s
Local efficiency	Rolandic Operculum L	Z = 0.5786	n.s
	Insula L	Z = 0.7832	n.s
	Temporal Mid L	Z = 1.2738	n.s
Clustering coefficient	Frontal Mid Orb R	Z = 1.6853	*p* = 0.0406
	Rolandic Operculum L	Z = 0.6179	n.s
	Insula R	Z = 0.9402	n.s
	Cuneus R	Z = 1.0720	n.s
	Paracentral Lobule L	Z = 0.5233	n.s
	Caudate_L	Z = 0.9640	n.s
Betweenness centrality	Frontal Mid Orb R	Z = 0.7991	n.s
	Lingual L	Z = 0.6765	n.s
	Parietal Sup R	Z = 0.7678	n.s
	Temporal Pole Sup L	Z = 0.0919	n.s

Orb, orbital part; Inf, inferior part; Mid, middle or median part; Sup, superior part, R, right; L, left; Post, posterior part; Ant, anterior part; FC, functional connectivity; Z: Fisher’s Z statistics; n.s., not significant. P-values surviving the Bonferroni correction are bolded.

#### ADE model

The test of ADE model applicability to node-level FC metrics highlighted a small number of ROIs, associated with specific features, influenced by ADE factors. The clustering coefficient was the feature with the highest number of ROIs (equal to 8.89% of the total) suitable for ADE modelling. Conversely, the strength of negative weights and betweenness centrality exhibited ADE effects in the minimum number of ROIs, accounting for 2.22% of the total. For each nodal feature, the ROIs for which the ADE model was suitable are listed in Table 3, together with the corresponding ADE coefficients and “broad-sense” heritability estimates. Significant correlation differences between MZ and DZ twins were observed only in the strength of positive weights in right middle temporal pole (Z = 1.66, *p* = 0.048).

**Table 3:** Location of ADE factor influences on node-level FC metrics reported for each ROI in which ADE model was applicable, and the corresponding ADE coefficients and “broad-sense” heritability estimates.

Node-level FC metric	ROI	A, D, E	${H}_{ADE}^{2}$
Nodal strength of positive weights	Precentral R	A = 7.86%, D = 8.64%, E = 83.50%	16.49%
	Lingual R	A = 14.15%, D = 8.75%, E = 77.10%	22.90%
	Occipital Mid R	A = 0.32%, D = 9.89%, E = 89.79%	10.20%
	Occipital Inf R	A = 24.22%, D = 13.74%, E = 62.03%	37.96%
	Fusiform L	A = 9.77%, D = 14.32%, E = 75.91%	24.08%
	Temporal Pole Sup L	A = 14.14%, D = 2.81%, E = 83.06%	16.94%
	Temporal_Pole_Mid_R	A = 1.71%, D = 59.75%, E = 38.54%	61.45%
Nodal strength of negative weights	Rectus R	A = 46.19%, D = 15.72%, E = 38.09%	61.91%
	Occipital Sup L	A = 11.26%, D = 20.81%, E = 67.93%	32.07%
Nodal degree	Amygdala L	A = 8.95%, D = 0.55%, E = 90.50%	9.49%
	Fusiform R	A = 2.54%, D = 43.39%, E = 54.07%	45.93%
	Temporal Pole Mid R	A = 25.43%, D = 12.20%, E = 62.37%	37.63%
Local efficiency	Calcarine L	A = 4.57%, D = 24.74%, E = 70.69%	29.30%
	Cuneus L	A = 4.69%, D = 13.27%, E = 82.05%	17.95%
	Lingual L	A = 5.28%, D = 22.96%, E = 71.77%	28.23%
	Paracentral Lobule L	A = 15.20%, D = 6.24%, E = 78.56%	21.44%
	Caudate L	A = 3.24%, D = 36.84%, E = 59.92%	40.08%
	Pallidum R	A = 16.28%, D = 37.77%, E = 45.95%	54.04%
	Temporal Pole Mid L	A = 11.31%, D = 26.39%, E = 62.29%	37.70%
Clustering coefficient	Insula L	A = 15.21%, D = 19.07%, E = 65.71%	34.28%
	ParaHippocampal L	A = 3.33%, D = 5.73%, E = 90.94%	9.06%
	Calcarine L	A = 4.06%, D = 24.12%, E = 71.82%	28.18%
	Cuneus L	A = 1.73%, D = 16.24%, E = 82.02%	17.98%
	Fusiform R	A = 27.02%, D = 11.14%, E = 61.83%	38.16%
	Pallidum R	A = 2.72%, D = 47.53%, E = 49.75%	50.24%
	Temporal Pole Mid L	A = 5.08%, D = 34.66%, E = 60.26%	39.74%
	Temporal Inf L	A = 28.22%, D = 23.97%, E = 47.82%	52.18%
Betweenness centrality	Putamen L	A = 43.42%, D = 8.84%, E = 47.74%	52.26%
	Thalamus R	A = 38.35%, D = 10.91%, E = 50.74%	49.25%

Orb, orbital part; inf, inferior part; mid, middle or median part; sup, superior part, r, right; l, left; post, posterior part; ant, anterior part.${H}_{ADE}^{2}$, “broad-sense” heritability estimate.

Overall, based on the ADE model coefficients and the related “broad-sense” heritability estimates, the environmental effects overcame additive and dominant genetic effects (E = 66.30 ± 15.51%, ${H}^{2}$= (A + D) = 33.70 ± 15.51%). The ADE-suitable node-level features that resulted strongly heritable (${H}^{2}$> 60%) were the strength of positive weights in right middle temporal pole and the strength of negative weights in right rectus. The latter was associated with the maximum “broad-sense” heritability estimate, equal to 61.91%. Predominant genetic effects (${H}^{2}$> 50%) were observed also for the betweenness centrality in left putamen, local efficiency in right pallidum, and clustering coefficient in both right pallidum and left inferior temporal gyrus.

#### ACE model

A few, localized node-level FC metrics met ACE modelling criteria. Among all features, local efficiency was influenced by ACE factors in the highest percentage (7.77%) of ROIs. On the contrary, the strength of negative weights and degree features showed ACE effects in the lowest number of ROIs, accounting for 3.33% of the total. Table 4 reports, for each node-level feature, the ROIs for which the ACE model was applied with the corresponding ACE coefficients and “narrow-sense” heritability estimates. No significant correlation differences between MZ and DZ twins were observed in any of the ACE suitable node-level features.

**Table 4:** Location of ACE factor influences on node-level FC metrics reported for each ROI in which ACE model was applicable, and the corresponding ACE coefficients and “narrow-sense” heritability estimates.

Node-level FC metric	ROI	A, C, E	${h}_{ACE}^{2}$
Nodal Strength of positive weights	Rectus L	A = 0.01%, C = 17.59%, E = 82.40%	0.01%
	Rectus R	A = 4.52%, C = 9.42%, E = 86.07%	4.52%
	Fusiform R	A = 16.25%, C = 28.42%, E = 55.32%	16.25%
	Postcentral R	A = 6.09%, C = 4.93%, E = 88.98%	6.09%
	Temporal_Inf_R	A = 31.16%, C = 4.75%, E = 64.09%	31.16%
Nodal Strength of negative weights	Frontal Sup R	A = 16.32%, C = 13.32%, E = 70.36%	16.32%
	Frontal Mid R	A = 17.75%, C = 4.53%, E = 77.73%	17.75%
	Fusiform L	A = 24.29%, C = 23.76%, E = 51.95%	24.29%
Nodal degree	Rectus L	A = 4.70%, C = 8.67%, E = 86.63%	4.70%
	Temporal Pole Sup L	A = 8.48%, C = 6.01%, E = 85.51%	8.48%
	Temporal Inf L	A = 11.25%, C = 2.28%, E = 86.47%	11.25%
Local efficiency	Insula R	A = 33.67%, C = 1.52%, 64,81%	33.67%
	Cingulum Post R	A = 30.95%, C = 1.55%, E = 67.50%	30.95%
	ParaHippocampal R	A = 3.95%, C = 13.28%, E = 82.78%	3.95%
	Occipital Sup L	A = 0.59%, C = 24.11%, E = 75.30%	0.59%
	Occipital Sup R	A = 5.11%, C = 0.77%, E = 94.11%	5.11%
	Fusiform R	A = 26.40%, C = 6.52%, E = 67.08%	26.40%
	Postcentral_L	A = 23.44%, C = 8.44%, E = 68.12%	23.43%
Clustering coefficient	Cingulum Post R	A = 31.93%, C = 1.42%, E = 66.65%	31.93%
	ParaHippocampal R	A = 6.04%, C = 9.16%, E = 84.77%	6.04%
	Occipital Sup L	A = 0.90%, C = 23.91%, E = 75.19%	0.90%
	Occipital Sup R	A = 5.63%, C = 0.48%, E = 93.89%	5.63%
	Postcentral L	A = 21.44%, C = 10.50%, E = 68.06%	21.44%
	Temporal Pole Mid R	A = 1.62%, C = 29.80%, E = 68.59%	1.62%
Betweenness centrality	Frontal Inf Triangular R	A = 10.26%, C = 2.28%, E = 87.46%	10.26%
	Cingulum Ant L	A = 33.58%, C = 0.04%, E = 66.38%	33.58%
	Calcarine L	A = 0.24%, C = 7.30%, E = 92.46%	0.24%
	Temporal Mid R	A = 13.68%, C = 10.47%, E = 75.84%	13.68%

Orb, orbital part; inf, inferior part; mid, middle or median part; sup, superior part, r, right; l, left; post, posterior part; ant, anterior part.${h}_{ACE}^{2}$: “narrow-sense” heritability estimate.

Accordingly, in all the ACE suitable node-level FC metrics, environmental effects overcame genetic ones ((C + E) = 86.06 ± 11.54%, ${h}^{2}$=A = 13.94 ± 11.54%). The highest genetic influence was observed on the local efficiency of right insula, whose “narrow-sense” heritability was estimated at 33.67%. Although unique environmental effects were predominant (> 50%) in all these metrics, shared environmental factors accounted for up to ~ 30% of variance. The features most affected by shared environment (> 20%) were, in descending order, the clustering coefficient of right middle temporal pole, the strength of positive weights of right fusiform gyrus, the local efficiency and clustering coefficient of left superior occipital cortex, and the strength of negative weights of left fusiform gyrus.

### Post-hoc RSN-level analysis

The results of the post-hoc analyses concerning the main RSNs are summarized in Table 5. For each RSN, the group average values of FC are reported in Figure S3. All the ROIs included in each RSN are reported as nodes in the figure, together with the strongest functional connections (with values above the 0.5 threshold). For each RSN, the percentages of network connections associated with epistatic effects or with genetic and environmental factors expressed by ACE/ADE twin models are reported. On average, ACE model explained the highest proportion of links in the auditory (40%), visual (~ 24%), and central executive (~ 9%) networks. Epistatic effects were prevailing in the connections of the motor (~ 11%) and salience (~ 9%) networks. Conversely, the basal ganglia network was characterized only by functional connections (~ 7%) influenced by the ADE model factors.

**Table 5:** Percentage of resting state network connections affected by epistatic effects or genetic and environmental influences expressed by ACE or ADE twin models.

RSN	Epistatic effect	ADE model	ACE model
SAL	8.79%	6.59%	6.59%
DMN	3.63%	5.04%	5.85%
CEN	7.67%	6.09%	9.25%
MOT	21.43%	7.14%	10.71%
VIS	4.40%	5.50%	24.18%
AUD	0%	6.67%	40.00%
BG	0%	6.67%	0

SAL, salience network; DMN, Default mode network; CEN, central executive network; MOT, sensorimotor network; VIS, visual network; AUD, auditory network; BG, basal ganglia network; RSN, Resting state network.

## Discussion

In our study, a cross-sectional rs-fMRI dataset from young pairs of twins was analyzed to gain insights into developmental genetic and environmental determinants of brain functional connectome. For the first time, these determinants were comprehensively explored using a multimodal analysis framework, based on multiple brain network features at multiple spatial scales (i.e., brain-, RSN-, node-, and link- levels) using multiple statistical approaches (including ADE and ACE models).

In line with prior knowledge, our preliminary evidence shows that brain FC is characterized by complex region- and feature-specific patterns of genetic-environmental effects throughout development. Of note, we found a good regional consistency of these effects across multiple spatial scales. Indeed, our findings suggest that frontotemporal FC is at least partially under genetic control, since epistatic and additive/dominant genetic influences were found both in selective frontotemporal connections and in the interactions of frontotemporal regions with the rest of the brain. Notwithstanding, we found an overall predominance of environmental influences on link-level and node-level FC features; unique environmental factors explained most of the inter-individual variability in the majority of FC features, but shared environment was also found to play a role on selective temporo-occipital connections.

If confirmed on larger samples, our preliminary results could inform on the physiological balance between genetic and environmental effects on multiple brain network features—at different spatial scales—in a wide developmental period. This knowledge might ultimately prove useful in understanding susceptibility and protective factors for mental illnesses that arise during this delicate life period.

### Developmental twin literature on the brain functional connectome

During the crucial developmental window, brain functional characteristics experience age-related trajectories under the effects of genetics and environment, which contribute towards the establishment of adult brain function. So far, only a limited number of twin studies have explored developmental determinants acting on rs-fMRI-derived FC measures. Nevertheless, the small number of twin studies and their heterogeneity in terms of sample characteristics, rs-fMRI data acquisition and processing protocols and statistical approaches have impeded the extraction of reproducible results.

In the panorama of developmental twin studies, our investigation is preliminary but innovative in different aspects. Firstly, the inclusion of link-level FC phenotypes has provided more accurate insights into subtle genetic and environmental influences. Secondly, analysis of multiple topological features has revealed feature-specific influences, which might underlie different neural processes. Likewise, the selection of the most adequate statistical approach for each phenotype has enabled a more tailored discrimination between additive/nonadditive genetic effects (at single/multiple loci) and shared/unique environmental effects. Of note, our results complement the sparse evidence from twin studies that explored rs-fMRI connectivity but in single developmental stages—from childhood^22,24^ through adolescence^16,36^ to young adulthood^21,37,38^—using heterogeneous graph metrics related to regional, RSN, or global features of the brain network.

### The role of genetics

A complex role of genetics on FC has been suggested by our pilot study, which includes epistatic effects, featured by the interaction of alleles at different loci of the chromosome, and additive and dominant effects, measuring the independent and interaction effects of alleles at a single locus.

Although previous researches suggest that nonadditive genetic variants influencing human complex traits are rare and often negligible compared to the additive genetic variation^39,40^, recent twin studies have also included nonadditive genetic in the overall phenotypic variation^4^. Thus, in classical twin studies we aren’t fully informed on the quantification of sources deviating from additive effect (i.e., C and D) and as a consequence such contributions cannot be simultaneously quantified and ADE/ACE twin models need to be alternatively chosen^41^. Interestingly Chen et al., 2015^41^ have suggested that nonadditive genetic components might often be masked by shared environmental factors, highlighting the potential risk of altered inhered genetic influences to the heritability estimates in small twin samples. Within this context, further investigations on which kind of deviations could arise besides a simple additive genetic model are needed.

Our findings suggest an epistatic genetic control over selective functional interactions of frontotemporal regions, including inferior and middle orbitofrontal gyrus, right inferior and middle temporal gyrus, and right amygdala. The greatest genetic effect was found in the connection between right middle temporal and right precentral gyrus. Epistasis mostly acted on node-level features of degree and connection strength, in regions mainly but not exclusively located in frontal and temporal lobes. Our post-hoc RSN-level analyses suggest epistatic influences on the FC within the motor network, being characterized by more than 20% of connections with these effects. Of note, the right frontotemporal FC was affected by additive and dominant genetic effects as well, as shown by the ADE model results. The ADE-suitable link- and node-level features associated with the highest broad-sense heritability (${H}^{2}$=(A + D) > 50%) were the FC link between right middle temporal pole and right middle cingulate cortex, as well as node-level features of strength of positive and negative weights in right middle temporal pole and right rectus, respectively. At the RSN level, the basal ganglia network connections were only suitable for ADE modelling, even if in a small percentage.

According to our link-level findings, the clusters of interconnected nodes in the sensorimotor, auditory, and salience RSNs -involving the amygdala, precentral gyrus, middle temporal cortex, and orbitofrontal cortex- were recently reported to be under strong genetic influence during childhood and adolescence^22,36^. Interestingly, the genetic control over the prefronto-temporal connections seems to originate during early infancy, as shown by Gao and colleagues^20^*,* and to persist throughout adulthood^38^.

At the regional level, the spatially sparse genetic influences that we observed on the node-level FC features -mainly connection strength and degree- have no precedents in the twin literature, due to the absence of studies that assessed the heritability of regional FC measures.

There have been researches analyzing the genetic and environmental influences on features reflecting local connectivity, but still extracted at the brain level. Moreover, their participants were either younger^24^ or older^21^. Since the impact of genes and environment on a specific brain feature was shown to be age-dependent^16,42,43^, their results are hardly comparable to ours. In this context, recent developmental investigations on brain functional networks have suggested age-dependent dynamic genetic-environmental influences on FC within cortical networks^16,20^. Nevertheless, whether these components dynamically influence FC during development is a matter of debate considering the absence of longitudinal twin studies from childhood through adolescence.

It should be remarked that, consistently with our RSN-level results, Fu et al., 2015^36^ showed stronger genetic influence on networks involved in sensorial processes, as sensorimotor and basal ganglia ones, than on networks involved in cognitive processes, as the default-mode, executive, and attention ones^37^. Other possible explanations for these differences may be related to distinct developmental trajectories of these networks. In fact, previous evidence has demonstrated that the sensory networks complete the development at very early ages^44,45^, whereas cognition-related networks continue their maturation throughout adolescence and early stage of adulthood^46^. Therefore, the faster development of primary sensory regions might reduce the capability of environment to shape sensory networks compared to executive ones. Nonetheless, multiple studies showed a considerable role of the common environment on the sensorimotor network^16,37^.

Our results suggest that some of the functional interactions within the salience network -especially involving the amygdala- are under genetic influence**.** In agreement with our findings, previous research on adolescent twins showed strong additive genetic influences over this network, which seem to be already present in the first years of life^16,20^. Notwithstanding, increasing body of evidence suggests that the amygdala-frontal FC is influenced by both genetic and environmental factors^22^. Of note, epigenetic studies on adolescent MZ twins demonstrated that unique life experiences can modify genetic expression, hormones, and amygdala-frontal interactions^23,47^. Our results should thus be interpreted while considering that our twin sample embraces different developmental stages and in turn the dynamics of genetic and environmental effects on FC.

### The role of environment

In our study, FC links and nodal features meeting ADE or ACE model criteria were found to be mostly affected by the unique environment. Unique environmental influences account for differences among twin couples during life, as well as measurement errors. Thus, our findings suggest an overall predominance of environmental relative to genetic effects on the developmental FC, at least in the features that were not influenced by epistasis.

However, these results might be carefully interpreted since measurement errors, defined as random and uncorrelated within twin pairs, could potentially influence the results related to unique environmental contribution^48^.

Furthermore, these environmental findings might seem in contrast with the strong heritability of the whole-brain FC-including global efficiency and mean clustering coefficient- that was reported across different developmental phases^20,21,24,38^. In this respect, it should be noted that our evidence does not result from global FC evidence, but from the integration of the effects observed on link- and node-level FC features. Moreover, our small sample has impeded the analysis of global FC features, which did not meet the criteria for ADE or ACE models’ application.

In this context, it is important to underline that although global metrics has been proposed to largely reflect effects at lower spatial scales^49^, specific characteristics of global topology may not be evident at finer spatial graphs’ levels^50^. This could suggest that global FC features may not be sensitive to specific node and link-level neurodevelopment and related influences. Moreover, the robustness of our findings regarding global FC features might have been influenced by the AAL parcellation or FC thresholding that have been chosen^51–53^.

Furthermore, AAL brain parcellation’s capability in representing functional systems is still under debate^54,55,56,57^. Nonetheless, AAL is the most widely used atlas employed to construct functional brain networks from the voxel level^58^, for heritability analysis on graph theoretical measures^21^ and for investigation of dynamic and non-dynamic FC patterns^56,57^. Therefore, the absolutely correct parcellation remains an enigma^58^.

Moreover, future analyses on larger independent samples coupled with the employment of multiple parcellations, or anatomo-functional parcellations schemes, are needed to respond to this open question and produce reproducible findings.

Evidence from the ACE analyses has shown selective FC links and nodes being influenced by the shared environment. Shared environmental influences at the level of FC links were shown to be predominant (C > 50%) in temporo-occipital connections, specifically in the links between the fusiform gyrus and the calcarine and middle/superior occipital cortices. Interestingly, common environmental effects acting on FC links within the visual network, or involving occipital regions, were previously reported^16,21^, whereas shared environmental effects concerning the fusiform gyrus were not previously indicated.

Our results suggest a role of common environment in shaping sensory networks, as the visual one, and occipital FC links. The lack of previous evidence supporting the effects on the fusiform gyrus could have been introduced by differences in the atlas choice or pre-processing approaches^16,59^.

Node-level FC features showed lower common environmental influences, which were mainly observed in the above regions. In particular, these effects explained more than 20% of the phenotypic variance (C > 20%) in the node-level features of clustering coefficient, specifically in the right middle temporal pole and left superior occipital cortex, local efficiency in the left superior occipital pole, and node strength in the fusiform gyrus. Based on our findings, environmental influences could play a non-negligible role in shaping the temporo-occipital architecture of functional brain communication during development. Twin literature evidence partially supports our findings, showing no effects of genes on the local clustering coefficient, indicating the level of local connectedness within the network^24^. It should be noticed that previous research has established the existence of genetic factors acting on these topological metrics, but at the whole-brain level, showing significant heritability of the global brain efficiency^21,24,38^ and clustering coefficient^21^. Nevertheless, these findings cannot be compared to ours due to the global vs. local spatial scale, and could not be supported by our study due to its limited sample size.

### Limitations

Several potential limitations of this study deserve a discussion. First, our twin sample was relatively small for accurate genetic modelling. The statistical power of genetic studies is influenced by the sample size, which affects the statistical significance of the intra-pair phenotypic correlation values. In our study, a non-negligible percentage of rMZ and rDZ estimates have not reached statistical significance (*p* < 0.05), thus impeding further analyses. Therefore, the heritability estimates were obtained from the Falconer’s formula^60^, whose formulation depends on the twin model applied. Although the Falconer’s formulation of heritability represents the simplest method of calculating the relative contribution of genetic effects, it generally provides a valid estimate of the phenotypic variation that is attributable to genetic factors^61^. However, a larger sample would have allowed fitting Structural Equation Modelling (SEM), offering a wide spectrum of straightforward hypothesis testing opportunities, including confounding and moderation effects on genetic and environmental estimates, as well as random measurements errors^62–64^. A larger sample would also have allowed us to reliably test for genetic effects of limited magnitude (i.e. broad heritability below 0.60) and for common environmental effects, for which the classical twin design is generally underpowered^65^. Nonetheless, over estimation of heritability may arise when fitting the model with multiple variables by employing a small sample size^62,66^. Nevertheless, each design suffers from specific issues, thus a trade-off between the available sample and robustness of genetic-environmental estimates should be reached.

Second, our analysis may be limited by the atlas’s choice that may have influenced the robustness of the results. Alternative methods, as the parallel use of multiple parcellation approaches or anatomical-functional parcellation schemes^67^ should be applied.

Furthermore, our study considered the fMRI time series extracted from 90 selected ROIs of the AAL atlas, since for most of the subjects the fMRI field of view did not cover the cerebellum and vermis. Therefore, the corresponding AAL ROIs were excluded from further FC analysis. Specifically, although the cerebellum and vermis are relatively small areas located deep in the brain and with low fMRI signal-to-noise ratio^67^, their exclusion from the FC analysis could have affected the results, since the anatomical location and the number of FC nodes have been reported to affect the properties of brain networks^68–71^. However, the inclusion of the majority of brain regions guarantees a robust FC network parcellation.

Third, in our study some FC features were computed after binarization of the FC matrix. The use of an arbitrary (correlation-based) 0.5 threshold might have influenced the results and their interpretation. A large instability of the network measures across binarization thresholds has been reported^72^. However, there is no consensus on the optimal threshold to be used and future analyses should explore multiple thresholds that allow evaluating how the network properties and the relative heritability estimates depend on the threshold’s choice.

Possible methodological concerns may also arise from the choice of the fMRI denoising pipeline, specifically for the lack of global signal regression (GSR) and ad-hoc motion censoring (e.g., using FD) that could have influenced the motions’ presence and signal-to-noise-ratio of BOLD signals. However, despite GSR being largely applied as pre-processing step for several favorable reasons^73^, its application is still a matter of debate in rs-fMRI analyses^74^, since recent evidence^75^ has hypothesized a neurophysiological basis for this global signal, suggesting that its regression may eliminate potential sources of neural activity. Likely, other concerns in our preprocessing steps may be referred to temporal filtering methods. Although we did not perform a bandpass filtering, recognized as standard practice for rs-fMRI processing in literature^76^, we removed both low-frequency drifts and high-frequency components before and during ICA denoising, respectively. Overall, there does not exist a general consensus on the optimal resting-state processing steps to be performed, which must be selected in the context of each analysis. In this respect, our pipeline provides an effective and robust denoising framework that was quantitatively evaluated on rs-fMRI data in our previous^32^ and current application.

Moreover, our study explored the determinants of brain FC using cross-sectional data, limiting the possibility of assessing the FC stability over development and impeding the analysis of age-dependent dynamic determinants of FC. In the absence of longitudinal information, alternative approaches that could be applied to a bigger cross-sectional twin sample might include the investigation of FC determinants on subsamples belonging to different developmental age sections. On the contrary, the wide developmental age range in our small sample might pose constraints to our cross-sectional evidence.

As last, although in our study nonadditive genetic effects have been associated to a prominent role on brain FC at several spatial scales, results related to these contributions need to be cautiously interpreted, since small twin sample provide inadequate power to significantly clearly separate deviating contributions (e.g., C and D) with respect to pure additivity^41^.

Moreover, more heritability studies testing for ADE model applicability may better highlight to what extent the variance in each brain FC graph metric could be attributable also to nonadditive genetic components.

## Conclusions

Our preliminary twin study has investigated the determinants of multi-scale functional brain network characteristics during development. Besides showing a predominance of unique environment, our results suggest the presence of genetic and common environmental influences on selective frontotemporal and temporo-occipital connections, respectively. The emergence of multiple (additive and nonadditive) genetic contributions to local connectivity features in specific brain regions remarks the utility of adopting a multi-model, multi-scale, and multi-feature approach. If reproduced on independent samples, this evidence might ultimately provide insights into genetic and environmental risk factors for developmental mental disorders.

## Supplementary Information

Supplementary Information.

## Data Availability

The clinical and MRI datasets supporting the current study have not been deposited in a public repository because of privacy and ethical restrictions, but are available from the corresponding author on request.
