# Supplementary material for: Environmental effects on brain functional networks in a juvenile twin population
Source: Sci Rep. 2023 Mar 9;13:3921. doi: 10.1038/s41598-023-30672-2 (PMC9998648; doi:10.1038/s41598-023-30672-2)
Supplement: Supplementary file 1 — Supplementary Information. [file 41598_2023_30672_MOESM1_ESM.pdf]

# Supplementary Materials for

Environmental effects on brain functional networks in a juvenile twin population.

Emma Tassi^, Eleonora Maggioni^, Maddalena Mauri, Corrado Fagnani, Nivedita Agarwal, Anna Maria Bianchi, Maria A. Stazi, Maria Nobile, Paolo Brambilla*

^ Equally contributing authors.

* Paolo Brambilla
Department of Neurosciences and Mental Health, Fondazione IRCCS Ca'Granda Ospedale Maggiore Policlinico, Milan, Italy.
Tel. +39 02-55032717.
**Email:** paolo.brambilla1@unimi.it

The document includes:

Supplementary **Figures S1, S2 and S3**.

Supplementary **Tables S1, S2 and S3**.

**Fig. S1. Schematic illustration of fMRI processing steps.**

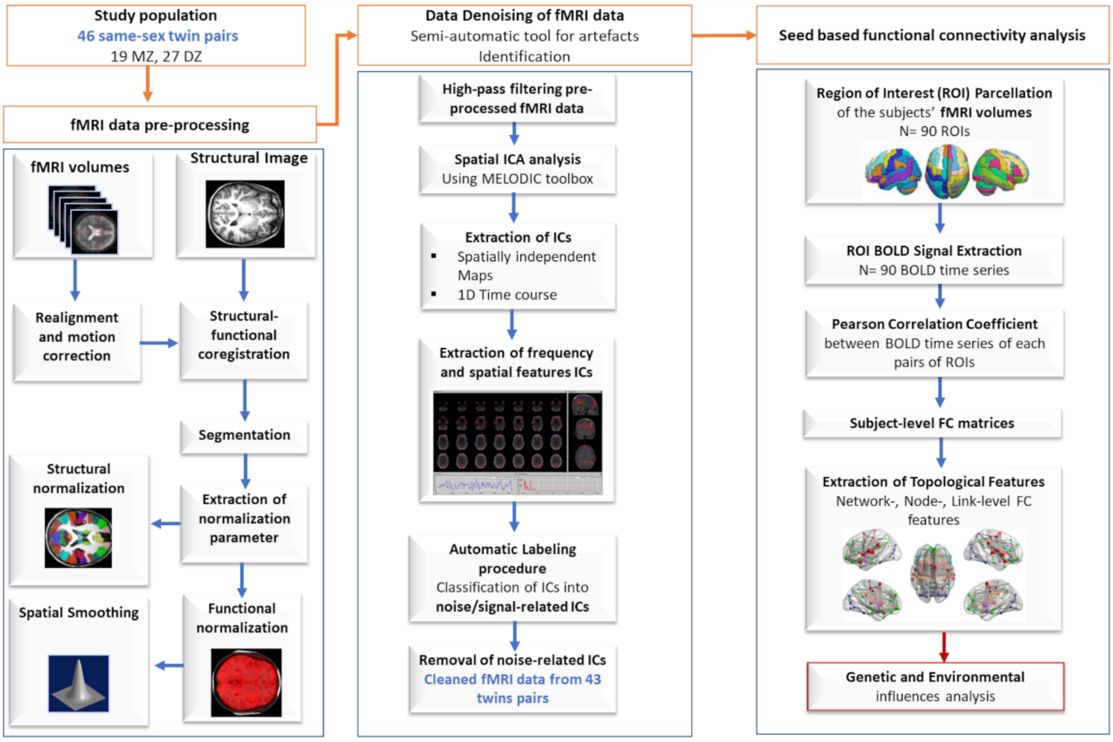


*The resting-state fMRI raw data from 46 same-sex twin pairs were pre-processed using SPM12. As follow, the data denoising of the pre-processed fMRI data was applied by the use of a semiautomatic tool for artefacts identification and subsequently the artefactual independent components (ICs) were regressed out from the data. At this step, the subjects associated with a percentage of noise-related ICs > 75% and their siblings were excluded from the analyses, resulting in a dataset composed of 43 twin pairs. Seed-based functional connectivity (FC) analyses were applied to the subject's fMRI volumes to extract the subject-level FC matrices based on the Automated Anatomic Atlas (AAL) parcellation. FC matrices were extracted by computing the Pearson correlation coefficients between BOLD time series. Multiple brain network features at different spatial scales (brain-, node- and link-level) were extracted from each subject's FC matrix and entered in the statistical analyses. Panels were assembled with Microsoft Power Point software (https://www.microsoft.com/it-it/microsoft-365/powerpoint) and exported using GIMP v.2-10 (https://www.gimp.org/).*

**Fig. S2. Barplots of $r_{MZ}$ and $r_{DZ}$ values for node-level FC metrics**.

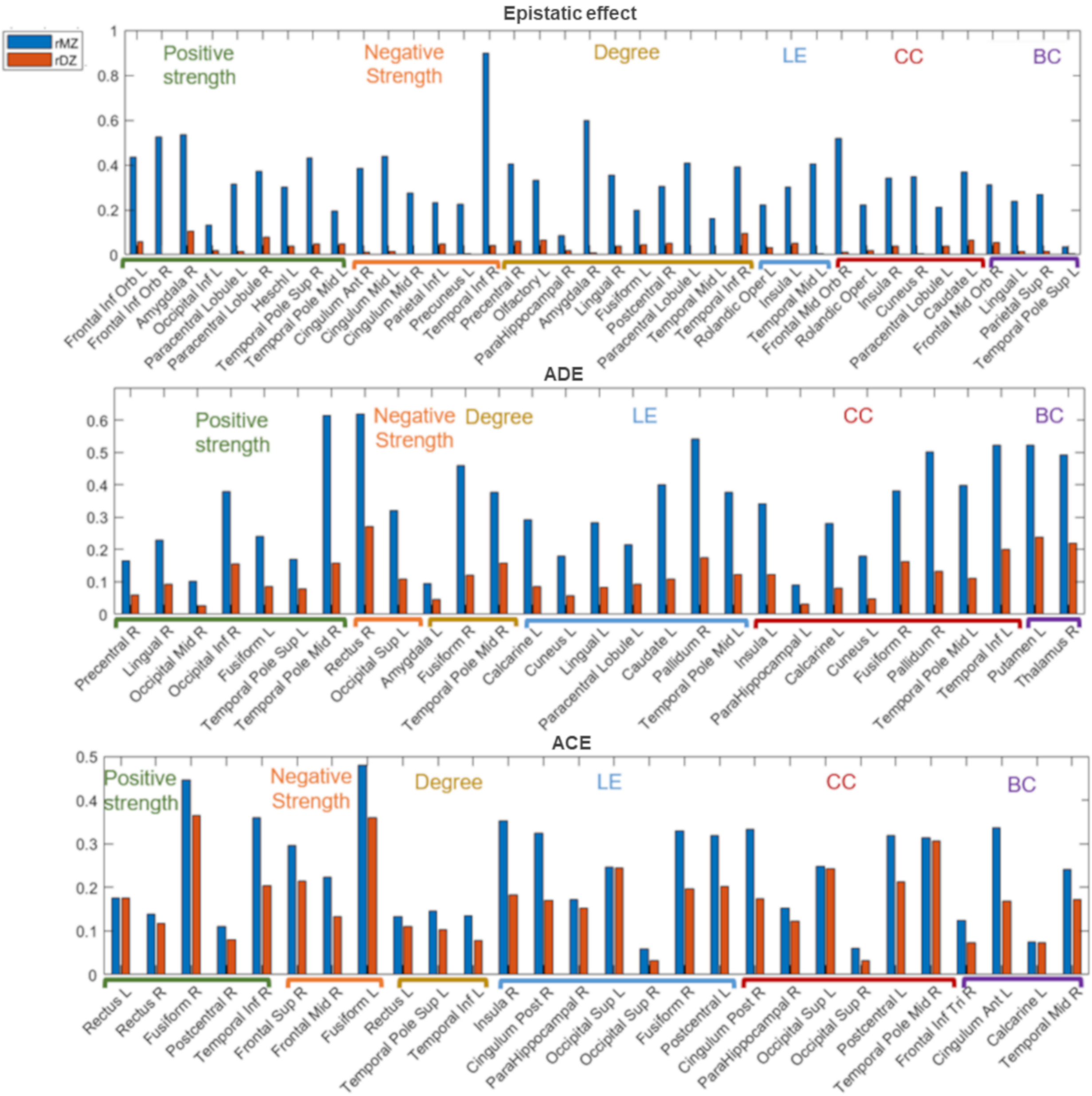


*$r_{MZ}$ and $r_{DZ}$ values associated with node-level FC metrics under epistasis (first panel), ADE effects (second panel), and ACE effects (third panel). $r_{MZ}$ values are illustrated in blue, while $r_{DZ}$ values are reported in red. $r_{DZ}$: intra-pair phenotypic correlation in DZ pairs. $r_{MZ}$: intra-pair phenotypic correlation in MZ pairs. Along the x-axis of each panel are reported the ROIs related to node-level FC metrics influenced by epistatic effect (first panel), ADE effects (second panel) and ACE effects (third panel). All the node-level metrics meeting the criteria for epistatic, ADE, or ACE effects are illustrated. Panels were assembled with Microsoft Power Point software (https://www.microsoft.com/it-it/microsoft-365/powerpoint) and exported using GIMP v.2-10 (https://www.gimp.org/).*

**Fig. S3. Group average FC of RSNs considered in group-level analysis.**

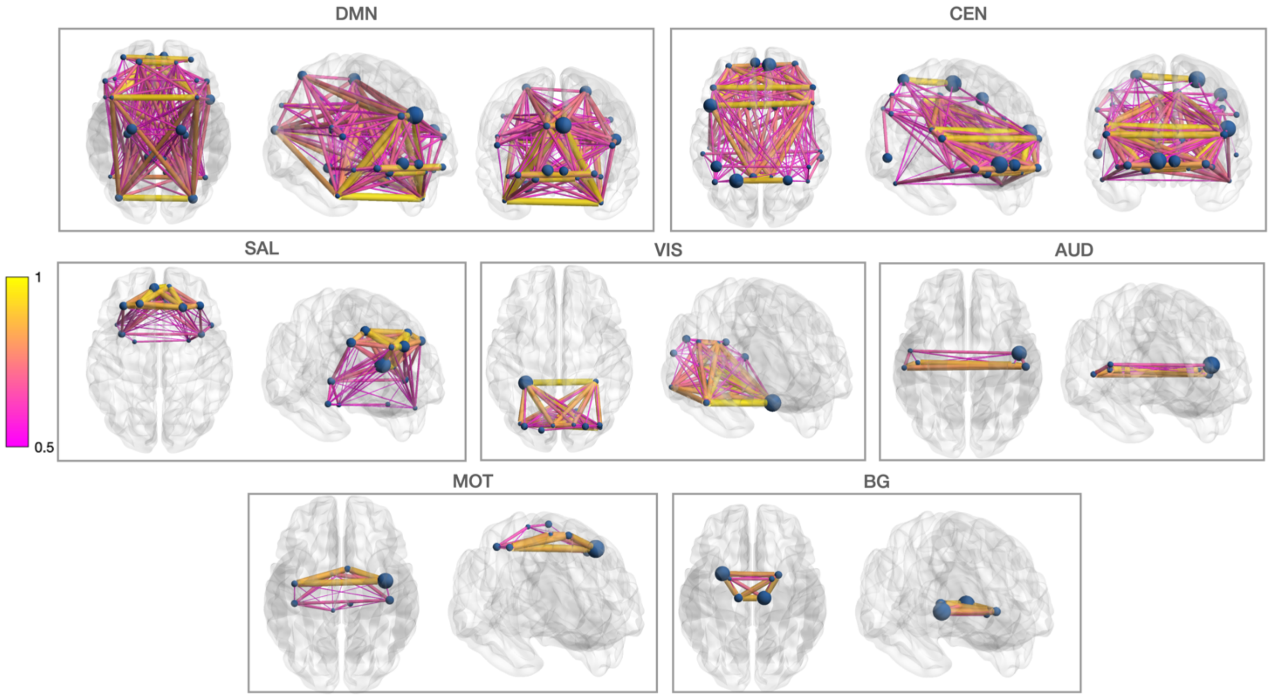


*All the **ROIs** and **group averaged FC links** above a threshold of 0.5 were reported for Default mode network (DMN), Central Executive network (CEN), Salience network (SAL), Visual network (VIS), Auditory network (AUD), Sensory motor network (MOT) and Basal ganglia (BG). 'The brain networks were visualized with the BrainNet Viewer (http://www.nitrc.org/projects/bnv/) [1] on R2019b software (http://www.mathworks.com). Panels were assembled with Microsoft Power Point software (https://www.microsoft.com/it-it/microsoft-365/powerpoint) and exported using GIMP v.2-10 (https://www.gimp.org/).*

**Table S1. Description of node-level functional connectivity (FC) metrics extracted for the analysis.**

| Node-level FC metrics | Description |
|---|---|
| Local efficiency | It describes the regional efficiency indicating the integration of each node. It represents the global efficiency computed on the neighbourhood of the node (extracted from binarized FC matrix). |
| Clustering coefficient | It describes the ability for functional segregation and efficiency of local information transfer (extracted from binarized FC matrix). |
| Degree | It represents number of links connected to the node indicating how many connections a node has to other nodes (extracted from binarized FC matrix). |
| Strength of weights | It indicates the average weight of connections each node has with other nodes. Calculated as the sum of the nodes associated to positive and negative weights (extracted from weighted FC matrix).<br>The *strength of positive weight* is calculated as the sum of the links connected to the nodes associated to a positive correlation with the others and represents the total positive strength of the node, and how much this node communicates in synchrony with the others.<br>The *strength of negative weight* is calculated as the sum of the links connected to the nodes associated to a negative correlation with the others and represents the total negative strength of the node, and how much this node communicates in asynchrony with the others. |
| Betweenees centrality | It indicates the fraction of all shortest paths in the network that contain a given node, if a node has a highest fraction of shortest paths, more communication across the network will pass through this node (extracted from binarized FC matrix). |

**Table S2. Description of Global-level functional connectivity (FC) metrics extracted for the analysis.**

| **Global FC metrics** | **Description** |
|---|---|
| Global efficiency | It is estimated as the average inverse shortest path length in the network, representing the integration over the whole brain network (extracted from binarized FC matrix). |
| Characteristic path length | It is calculated as the average shortest path length in the network and representing how "efficiently" the brain is connected (extracted from binarized FC matrix). |
| Degree | It represents the average degree in the network (see Table S1, extracted from binarized FC matrix). |
| Density | It indicates the fraction of present connections out of all possible connections, indicate how densely connected is a graph (extracted from binarized FC matrix). |
| Louvain modularity | It represents the modularity of a network and quantifies the "strength" of partition of a network into modules (also called communities and clusters). Louvain method is an efficient method to identify communities in large network. The method is a greedy optimization that attempts to optimize the modularity of a partition of a network by: firstly, it looks for small communities optimizing modularity locally, then it aggregates nodes belonging to the same community and builds a new network whose nodes are the communities (extracted from binarized FC matrix). |

**Table S3. Automated Anatomical Labeling (AAL) atlas regions included in the resting state networks (RSNs).**

| RSN | AAL regions |
|---|---|
| Salience Network | Superior frontal gyrus, dorsolateral<br>Middle frontal gyrus<br>Insula<br>Amygdala<br>Temporal pole: superior temporal gyrus<br>Anterior cingulate and paracingulate gyri<br>Superior frontal gyrus, medial |
| Default Mode Network | Anterior cingulate and paracingulate gyri<br>Superior frontal gyrus, medial<br>Median cingulate and paracingulate gyri<br>Posterior cingulate gyrus<br>Hippocampus<br>Parahippocampal gyrus<br>Middle occipital gyrus<br>Temporal pole: middle temporal gyrus<br>Superior frontal gyrus, orbital part<br>Middle frontal gyrus, orbital part<br>Inferior frontal gyrus, orbital part<br>Superior frontal gyrus, medial part<br>Angular gyrus<br>Precuneus<br>Inferior frontal gyrus, opercular part<br>Inferior frontal gyrus, triangular part |
| Central Executive Network | Superior frontal gyrus, orbital part<br>Middle frontal gyrus, orbital part<br>Inferior frontal gyrus, orbital part<br>Superior frontal gyrus, medial part<br>Angular gyrus<br>Precuneus<br>Inferior frontal gyrus, opercular part<br>Inferior frontal gyrus, triangular part<br>Superior parietal gyrus<br>Inferior parietal, supramarginal and angular gyri<br>Supramarginal gyrus<br>Caudate nucleus<br>Middle temporal gyrus<br>Inferior temporal gyrus |
| Motor Network | Precentral gyrus<br>Postcentral gyrus<br>Supplementary motor area<br>Paracentral lobule |
| Visual Network | Cuneus<br>Lingual gyrus<br>Calcarine fissure and surrounding cortex<br>Superior occipital gyrus<br>Middle occipital gyrus<br>Inferior occipital gyrus<br>Fusiform gyrus |
| Auditory Network | Heschl gyrus<br>Superior temporal gyrus<br>Rolandic operculum<br>Thalamus |
| Basal Ganglia Network | Thalamus<br>Lenticular nucleus, putamen<br>Lenticular nucleus, pallidum |

*RSN, Resting-state network.*

## References

1. *Xia, M., Wang, J. & He, Y. BrainNet Viewer: A Network Visualization Tool for Human Brain Connectomics. PLoS ONE* ***8****, e68910 (2013).*
